# Supplementary material for: Impact of Extraskeletal Metastases on Skeletal-Related Events in Metastatic Castration-Resistant Prostate Cancer with Bone Metastases
Source: Cancers (Basel). 2020 Jul 24;12(8):2034. doi: 10.3390/cancers12082034 (PMC7463577; doi:10.3390/cancers12082034)
Supplement: Supplementary file 1 [file cancers-12-02034-s001.pdf]

Table S1. Bone biomarkers according to metastatic compartment.

Characteristic		BOM	BESM	*p*-Value
**Baseline assessment**				
Uncorrected uNTX, nmol BCE/mmol creatinine				
	Median	513	434	0.044
	P25–P75	246–1214	209–1040	
	Min.–max.	63–18,571	63–28,571	
Corrected uNTX, nmol BCE/mmol creatinine				
	Median	48	48	0.277
	P25–P75	27–100	25–85	
	Min.–max.	4–3 904	4–1 786	
Corrected NTX, *n* (%)				
	High	628 (42.6)	131 (40.1)	0.410
	Normal	848 (57.5)	196 (59.9)	
bALP, ng/mL				
	Median	31	29	0.899
	P25–P75	17–74	17–75	
	Min.–max.	4–1 363	0–1941	
bALP, *n* (%)				
	High	1012 (66.9)	221 (66.2)	0.789
	Normal	500 (33.1)	113 (33.8)	
**3-month assessment**				
Uncorrected uNTX, nmol BCE/mmol creatinine				
	Median	71	77	0.796
	P25–P75	63–162	63–161	
	Min.–max.	63–17,984	63–5802	
Corrected uNTX, nmol BCE/mmol creatinine				
	Median	9	10	0.428
	P25–P75	6–21	6–21	
	Min.–max.	2–1754	3–556	
Corrected uNTX, *n* (%)				
	High	113 (9.1)	19 (7.4)	0.402
	Normal	1136 (90.9)	237 (92.6)	
Corrected uNTX normalization[1], *n* (%)				
	Yes	364 (80.2)	77 (85.6)	0.234
	No	90 (19.8)	13 (14.4)	
bALP, ng/mL				
	Median	20	20	0.916
	P25–P75	11–67	11–61	
	Min.–max.	3–1461	4–640	
bALP, *n* (%)				
	High	622 (50.1)	125 (49.4)	0.845
	Normal	620 (49.9)	128 (50.6)	
bALP normalization[1], *n* (%)				
	Yes	236 (29.0)	56 (32.9)	0.310
	No	577 (71.0)	114 (67.1)	

ALP, alkaline phosphatase; BOM, bone-only metastases; BESMD, bone and extra-skeletal metastases; uNTX, urinary N-terminal telopeptide; P25, Percentile 25; P75, Percentile 75.[1]If elevated at baseline. Cut-off for normal uNTX <64 nmol BCE/mmol creatinine; cut-off for normal bALP <22 ng/mL, as per R. Coleman 2011 CROH [13].
